# Ethanol lock therapy for salvage of infected tunnelled haemodialysis catheters: a randomised controlled trial

**DOI:** 10.1093/ckj/sfag013

**Published:** 2026-01-19

**Authors:** Hari Prasad M K, Lalit K Pursnani, Himansu Sekhar Mahapatra, Muthukumar Balkrishnan, Renju Binoy, Vipin Dev, Varuna Yadav, Disha Arora

**Affiliations:** Department of Nephrology, Atal Bihari Vajpayee Institute of Medical Sciences & Dr Ram Manohar Lohia Hospital, New Delhi, India; Department of Nephrology, Atal Bihari Vajpayee Institute of Medical Sciences & Dr Ram Manohar Lohia Hospital, New Delhi, India; Department of Nephrology, Atal Bihari Vajpayee Institute of Medical Sciences & Dr Ram Manohar Lohia Hospital, New Delhi, India; Department of Nephrology, Atal Bihari Vajpayee Institute of Medical Sciences & Dr Ram Manohar Lohia Hospital, New Delhi, India; Department of Nephrology, Atal Bihari Vajpayee Institute of Medical Sciences & Dr Ram Manohar Lohia Hospital, New Delhi, India; Department of Nephrology, Atal Bihari Vajpayee Institute of Medical Sciences & Dr Ram Manohar Lohia Hospital, New Delhi, India; Department of Nephrology, Atal Bihari Vajpayee Institute of Medical Sciences & Dr Ram Manohar Lohia Hospital, New Delhi, India; Department of Nephrology, Atal Bihari Vajpayee Institute of Medical Sciences & Dr Ram Manohar Lohia Hospital, New Delhi, India

**Keywords:** catheter-related infection, ethanol lock, tunnelled catheter

## Abstract

**Background:**

Tunnelled catheter–related bloodstream infections (CRBSIs) in haemodialysis (HD) are challenging to manage due to biofilm formation. Ethanol lock therapy (ELT) has demonstrated potential as an adjunct to antibiotics for catheter salvage, but robust evidence is limited.

**Methods:**

We conducted a single-centre, open-label, randomised controlled trial of adult HD patients with suspected or confirmed CRBSI. Patients received either 70% ELT plus intravenous antibiotics or intravenous antibiotics alone. Primary outcome was catheter salvage at day 7. Secondary outcomes included recurrence at day 60, catheter survival and adverse events.

**Results:**

Eighty-four patients were randomised (42 per arm). Coagulase-negative *Staphylococcus* was the most common pathogen (34.5%). Early catheter salvage was higher with ELT (78.6% versus 57.1%; *P* = .035). Recurrence was lower with ELT at day 60 (20.5% versus 53.7%; *P* = .002). The median catheter survival was longer (15 versus 8 days), although not statistically significant (*P* = .283). Fever resolution by day 7 was significantly higher in the ELT group compared with controls (64.3% versus 35.7%; *P* = .009). Adverse events were infrequent and mild. In multivariate analysis, higher serum albumin was independently associated with an increased likelihood of catheter salvage [odds ratio (OR) 2.43, *P* = .038], while longer dialysis vintage (OR 0.90, *P* = .035) and *Pseudomonas* infection (OR 0.05, *P* = .014) were associated with reduced salvage rates.

**Conclusion:**

Adjunctive ELT improved early catheter salvage and reduced recurrence without significant adverse effects. These findings support its use as part of salvage protocols in tunnelled catheter infections.

## GRAPHICAL ABSTRACT

**GRAPHICAL ABSTRACT:**
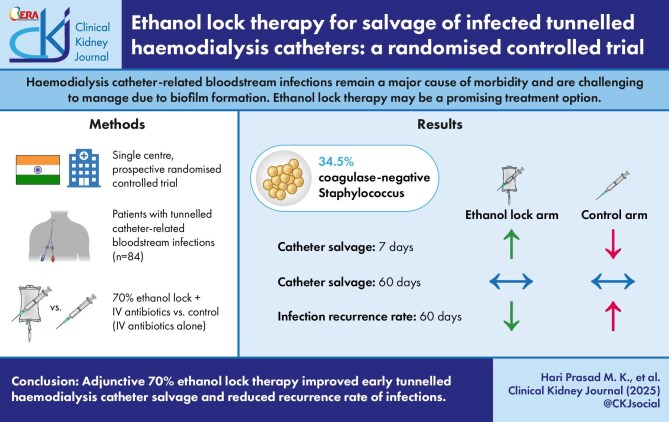

KEY LEARNING POINTS **What was known:**

- Ethanol lock therapy (ELT) improves early catheter salvage when used as an adjunct to systemic antibiotics in tunnelled catheter–related bloodstream infections.

**This study adds:**

- The early benefit of ELT does not translate into a significant improvement in long-term catheter survival.

**Potential impact:**

- Higher serum albumin improves catheter salvage while longer dialysis vintage and *Pseudomonas* infection predict salvage failure.

## Introduction

Catheter-related bloodstream infections (CRBSIs) remain among the most serious complications of tunnelled central venous catheters (CVCs), with reported incidences ranging from 2 to 5 per 1000 catheter days [1]. Vascular access choice follows a well-established hierarchy, with the arteriovenous fistula (AVF) as the preferred access owing to its durability and lowest infection risk. When AVF creation is not feasible, an arteriovenous graft (AVG) may be used. CVCs are reserved as a last resort or as a temporary bridge while awaiting AVF maturation.

However, CVCs are associated with a high burden of complications, including infection, thrombosis, central vein stenosis and increased morbidity and mortality. Once established, infections are difficult to eradicate due to biofilm formation within the catheter lumen, which protects pathogens from both systemic antibiotics and host immune responses. Standard management involves intravenous (IV) antibiotics and, if unsuccessful, catheter removal and replacement [2]. However, immediate catheter removal is not always feasible, especially in patients with limited vascular access or those awaiting AVF maturation. In such cases, catheter salvage strategies become essential to reduce the morbidity associated with repeated access procedures.

Antimicrobial lock solutions (ALSs) have been explored as adjuncts to systemic therapy, aiming to eliminate intraluminal microbial colonization, but are cost inefficient and expensive. Ethanol lock therapy (ELT), typically using 70% ethanol, has gained attention due to its broad-spectrum antimicrobial effect, ability to penetrate biofilms, cost efficiency and minimal risk of promoting antimicrobial resistance [3].

The Healthy Cath Trial was among the first randomized trials to demonstrate that ethanol locks reduced CRBSI incidence compared with heparin locks [4]. Subsequent studies, including that by Gang *et al.* [5], have shown that combining ELT with systemic antibiotics leads to significantly improved catheter salvage and prolonged tunnelled CVC survival. Case reports by Akhil *et al.* [6] also demonstrated successful salvage using ethanol locks in infections refractory to antibiotics alone. *In vitro* studies, such as that by Alonso *et al.* [7], have confirmed ethanol’s ability to reduce metabolic activity and biofilm biomass in CRBSI pathogens. Despite promising data, most studies to date have been observational, single-centre or lacked randomization. Systematic reviews and trials have emphasized the potential utility of ELT while underscoring the need for higher-quality evidence [8, 9].

This randomized controlled trial was conducted to determine whether ethanol locking, as an adjunct to IV antibiotics, improves catheter salvage rates in patients with tunnelled catheter–related bloodstream infections. Further, recurrence, microbiological profiles, resistance patterns and mortality, with the ultimate goal of refining salvage strategies in clinical nephrology, have also been studied.

## Materials and methods

### Study design and setting

We conducted a prospective, open-label, randomized controlled trial between August 2023 and December 2024 at the Department of Nephrology in a tertiary care hospital in Delhi after obtaining ethics clearance.

### Sample size

The sample size was based on expected catheter salvage rates of 89% with ELT and 41% with antibiotics alone (power 80%, alpha 0.05), yielding 31 per arm. To account for attrition, 76 patients were planned [5].

### Inclusion and exclusion criteria

Eligible participants were adults (≥18 years) on maintenance haemodialysis (HD) with a tunnelled catheter–related bloodstream infection, defined by the presence of fever (>38°C) or intradialytic chills/rigors without another identified source of infection. Exclusion criteria included acute kidney injury, fungal or mycobacterial bloodstream infection, refractory shock requiring vasopressors, pregnancy, documented alcohol allergy or tunnel or exit site infection at presentation.

### Definitions

CRBSI encompasses both microbiologically confirmed and clinically suspected infections in symptomatic patients without another identifiable source. It requires isolation of the same organism from both a semiquantitative catheter tip culture (>15 CFU/segment) and blood culture. Clinically refers to resolution of symptoms following antibiotic therapy or catheter removal, even in the absence of laboratory confirmation. Exit site infection included hyperaemia, induration and/or tenderness up to 2 cm from the catheter exit site and may be associated with fever and purulent drainage from the exit site. Tunnel infection included tenderness, hyperaemia and/or induration that extends >2 cm from the exit site and along the subcutaneous tunnel.

### Data collection

Baseline data included demographics, comorbidities, dialysis vintage and catheter characteristics. Laboratory assessments [complete blood count, C-reactive protein (CRP), procalcitonin (PCT), serum albumin] were performed on days 0, 5, 30 and 60 using standardized analysers. Blood cultures were drawn from both catheter ports and peripheral veins at enrolment and on days 5, 15, 30 and 60 and processed using the BD BACTEC FX40 system (BD, Franklin Lakes, NJ, USA) with standard antimicrobial susceptibility testing.

### Randomization and interventions

Participants were randomized 1:1 to one of two arms using computer-generated randomization. The intervention group received 70% ELT plus IV antibiotics and the control group received IV antibiotics alone.

The ethanol lock solution was prepared aseptically by mixing 99.99% ethanol with sterile distilled water and 1000 U/ml heparin, instilled daily into the catheter lumens for a dwell time of 4 hours over 7 days. Systemic antibiotics included empirical vancomycin (1 g IV every 48 h) and ceftazidime (1 g IV every 24 h), adjusted per microbial susceptibility.

### Rationale for intervention protocol

ELT was administered once daily for 7 days with a 4-hour dwell time to achieve rapid intraluminal sterilisation during the acute phase of infection, when biofilm burden is highest. The duration was limited to 7 days due to the potential wear effect of ethanol on catheter integrity and because continuation beyond 1 week provides no additional benefit in local sterilisation, as catheters are typically removed within this time frame according to Kidney Disease Outcomes Quality Initiative recommendations. Systemic antibiotics were continued for 14 days and extended to 21 days for *Staphylococcus aureus* or *Pseudomonas*, in line with guideline recommendations. This strategy balanced local control of biofilm with adequate systemic coverage for bloodstream infection.

### Laboratory evaluation

Blood samples were collected on days 0, 5, 30 and 60 for hemogram, PCT, quantitative CRP and serum albumin. Automated systems included Sysmex XN-1000 (haematology; Kobe, Japan), Roche Cobas e411 (CRP, PCT; Basel, Switzerland), and Beckman Coulter AU5800 (albumin; Indianapolis, IN, USA). Blood cultures were drawn at fever onset, using aerobic and anaerobic BACTEC bottles, from catheter ports and peripheral sites. Cultures were incubated in the BD BACTEC FX40 system. Positive cultures underwent Gram stain, subculture and antimicrobial susceptibility testing using standard techniques, including disc diffusion and automated methods.

#### Outcome measures

Catheter salvage was defined by resolution of fever and intradialytic symptoms and the absence of culture positivity by day 7. Antibiotic therapy was continued for 14 days, extended to 21 days in methicillin-resistant *Staphylococcus aureus* (MRSA) or *Pseudomonas* infections. Failure criteria included persistent symptoms or positive cultures by day 7, tunnel/exit site infection or systemic complications. Long-term outcomes included relapse or reinfection up to day 60.

#### Follow-up

Patients were clinically assessed and sampled on days 7, 15, 30 and 60. Blood cultures were repeated on days 5, 15, 30 and 60 to assess microbial clearance, relapse or reinfection.

### Statistical analysis

Analyses were performed using SPSS version 25.0 (IBM, Armonk, NY, USA). Normality was tested by the Kolmogorov–Smirnov method. Continuous variables were summarized as median [interquartile range (IQR)] and compared using Mann–Whitney U tests. Categorical variables were presented as counts (percentages) and compared using chi-squared or Fisher’s exact tests. Kaplan–Meier survival curves and logrank tests evaluated catheter survival. Logistic regression models (univariate and multivariate) identified predictors of catheter salvage, with odds ratios (ORs) and 95% confidence intervals (CIs) reported. Two-sided *P*-values <.05 were considered statistically significant.

## Results

### Study population

**Figure 1:**
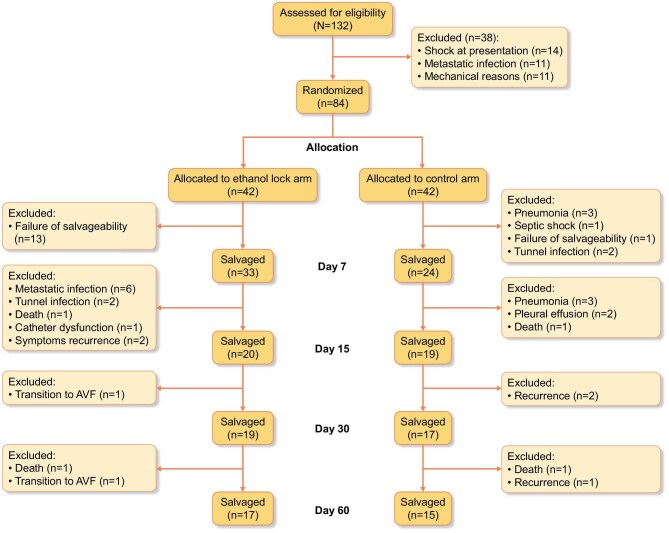
CONSORT diagram.

As shown in Fig. 1, a total of 132 patients with CRBSIs were screened for eligibility. Of these, 28 were excluded for not meeting inclusion criteria or declining consent. The remaining 84 patients were randomized equally into two groups: 42 patients in the ethanol lock arm and 42 in the control arm. All enrolled patients completed follow-up and were included in the final analysis.

### Baseline characteristics

All 84 patients were dependent on a tunnelled CVC because an AVF was not possible or not available at the time of enrolment. As the primary objective of our study was to assess catheter salvage, we did not further categorize the individual reasons for AVF unsuitability.

Baseline demographic and clinical characteristics were well balanced between the two groups (Table 1). The median age of participants was 50.5 years (IQR 37.75–60), with no significant difference between groups (*P* = .519). Females constituted 61.9% of the total cohort. The most common underlying cause of chronic kidney disease was diabetic nephropathy (39.3%), followed by chronic glomerulonephritis (33.3%). Comorbidities were similarly distributed between groups, with hypertension (47.6%) and diabetes mellitus (36.9%) being most prevalent. The right internal jugular vein was the most common catheter insertion site (61.9%). There were no significant differences in dialysis vintage (*P* = .054) or duration of catheter use (*P* = .053).

**Table 1:** Comparison of baseline characteristics between the ELT arm and control arm.

Baseline characteristics	Total	ELT arm (*n* = 42)	Control arm (*n* = 42)	*P*-value
Female, *n* (%)	52 (61.90)	25 (59.52)	27 (64.29)	.653^c^
Age (years), median (IQR)	50.5 (37.75–60)	54 (38–60.75)	46.5 (38–55.75)	.519^a^
Basic disease, *n* (%)				
Diabetic kidney disease	33 (39.29)	16 (38.10)	17 (40.48)	.977^b^
Hypertensive nephropathy	6 (7.14)	3 (7.14)	3 (7.14)	
CGN	28 (33.33)	14 (33.33)	14 (33.33)	
CTID	9 (10.71)	6 (14.29)	3 (7.14)	
Renovascular	2 (2.38)	1 (2.38)	1 (2.38)	
Acute cortical necrosis	1 (1.19)	0 (0)	1 (2.38)	
ADPKD	1 (1.19)	0 (0)	1 (2.38)	
CAKUT	2 (2.38)	1 (2.38)	1 (2.38)	
Comorbidities, *n* (%)				
None	2 (2.38)	0 (0)	2 (4.76)	.257^b^
Diabetes mellitus	31 (36.90)	15 (35.71)	16 (38.10)	
Hypertension	40 (47.62)	19 (45.24)	21 (50)	
Other	11 (13.10)	8 (19.05)	3 (7.14)	
Catheter site, *n* (%)				
Right internal jugular	52 (61.90)	24 (57.14)	28 (66.67)	.747^b^
Left internal Jugular	20 (23.81)	10 (23.81)	10 (23.81)	
Right femoral	5 (5.95)	3 (7.14)	2 (4.76)	
Left femoral	1 (1.19)	1 (2.38)	0 (0)	
Right external jugular	6 (7.14)	4 (9.52)	2 (4.76)	
Prior CRBSI, *n* (%)	25 (29.76)	14 (33.33)	11 (26.19)	.349^b^
Prior antibiotics, *n* (%)				
None	67 (79.76)	35 (83.33)	32 (76.19)	.162^b^
Ceftazidime	10 (11.90)	6 (14.29)	4 (9.52)	
Ceftazidime plus vancomycin	7 (8.33)	1 (2.38)	6 (14.29)	
Duration of catheter insertion (months), median (IQR)	4 (2–6)	4 (2.25–7.5)	3 (2–4.75)	.053^a^
Dialysis vintage (months), median (IQR)	6 (4–12)	12 (4.625–12)	6 (3–12)	.054^a^

a Mann–Whitney test.

b Fisher’s exact test.

c Chi-squared test.

The baseline characteristics were well-balanced between the ethanol lock and control arms, with no statistically significant differences in demographics, comorbidities, catheter insertion sites, prior infections or microbiological profile (*P* > .05 for all comparisons). This ensures that any observed differences in outcomes are attributable.

### Laboratory and microbiological parameters

Baseline laboratory parameters were largely comparable between groups (Supplementary Table 1). Median haemoglobin, leucocyte counts, platelet counts, CRP and albumin levels did not differ significantly. However, moderate PCT elevation (2–5 ng/ml) was more frequent in the control arm (16.7% versus 2.4%; *P* = .032), although most patients in both groups had markedly elevated PCT levels (>10 ng/ml).

The microbiological spectrum of baseline blood cultures was similar across groups (Table 2). Coagulase-negative *Staphylococcus* (CONS; 34.5%) was the most common pathogen, followed by *Klebsiella* (19.1%), *S. aureus* (13.1%) and *Pseudomonas* species (11.9%). Blood cultures were sterile in 15.5% of patients. The calculated CRBSI rate was 6.21 per 1000 catheter days.

**Table 2:** Comparison of baseline culture between ELT and control arm.

Baseline organism	Total	ELT arm (*n* = 42)	Control arm (*n* = 42)	*P*-value
Sterile, *n* (%)	13 (15.48)	7 (16.67)	6 (14.29)	.949^a^
CONS, *n* (%)	29 (34.52)	15 (35.71)	14 (33.33)	
*Staphylococcus aureus, n* (%)	11 (13.10)	4 (9.52)	7 (16.67)	
*Klebsiella, n* (%)	16 (19.05)	9 (21.43)	7 (16.67)	
*Pseudomonas, n* (%)	10 (11.90)	5 (11.90)	5 (11.90)	
*Enterobacter, n* (%)	1 (1.19%)	1 (2.38)	0 (0)	
*Escherichia coli, n* (%)	1 (1.19)	0 (0)	1 (2.38)	
*Citrobacter, n* (%)	1 (1.19)	0 (0)	1 (2.38)	
*Acinetobacter, n* (%)	2 (2.38)	1 (2.38)	1 (2.38)	

a Fisher’s exact test. The distribution of a similar organism was seen between the two groups (*P* = .949), with CONS being the most frequently isolated pathogen, followed by *Klebsiella pneumoniae, Staphylococcus aureus* and *Pseudomonas aeruginosa*. The proportion of sterile cultures was also comparable, ensuring that the microbiological profiles at baseline did not bias the assessment of catheter salvageability.

**Figure 2:**
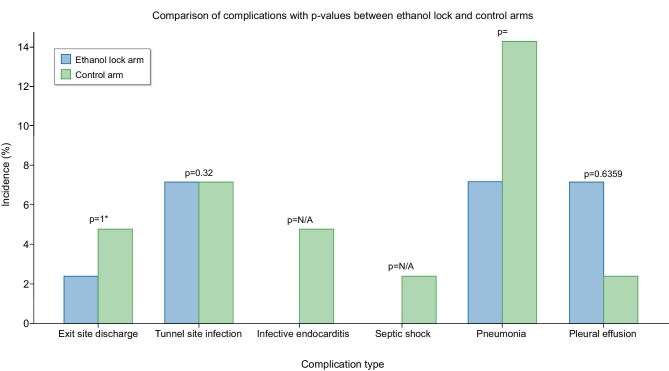
Complications of catheter-related infections.

By day 7, fever persisted in a significantly smaller proportion of patients in the ELT group (35.7% versus 64.3%; *P* = .009). Differences at subsequent follow-up points were not statistically significant (Table 3). Intradialytic chills and dyspnoea also improved in both groups, with no significant differences after day 15. Serious complications were numerically more common in the control arm but not significant (Fig. 2).

**Table 3:** Comparison of fever, intradialytic chills and dyspnoea between the ELT and control arms.

	Fever				Intradialytic chills				Dyspnoea			
Follow-up	Total (n = 84)	ELT arm (n = 42)	Control arm (n = 42)	*P*-value	Total (n = 84)	ELT arm (n = 42)	Control arm (n = 42)	*P*-value	Total (n = 84)	ELT arm (n = 42)	Control arm (n = 42)	*P*-value
Day 0	84 (100)	42 (100)	42 (100)	NA	84 (100)	42 (100)	42 (100)	NA	18 (21.43)	5 (11.90)	13 (30.95)	0.033^†^
Day 7	42 (50)	15 (35.71)	27 (64.29)	.009^†^	42 (50)	18 (42.86)	24 (57.14)	.19^†^	29 (34.52)	13 (30.95)	16 (38.10)	0.491^†^
Day 14	13 (15.66)	6 (14.63)	7 (16.67)	.799^†^	9 (10.84)	4 (9.76)	5 (11.90)	1^*^	10 (12.05)	5 (12.20)	5 (11.90)	0.968^†^
Day 30	2 (2.41)	2 (4.88)	0 (0)	.241^*^	5 (6.02)	3 (7.32)	2 (4.76)	.676^*^	1 (1.20)	0 (0)	1 (2.38)	1^*^
Day 60	3 (3.66)	1 (2.44)	2 (4.88)	1^*^	7 (8.54)	4 (9.76)	3 (7.32)	1^*^	0 (0)	0 (0)	0 (0)	NA

Values are presented as *n* (%).

* Indicates Fisher's exact test.

† Indicates Chi-squared test.

Local catheter site infections were infrequent and comparable between groups, with low rates of tunnel or exit-site infections observed during follow-up.

### Primary and secondary outcomes

Catheter salvage at day 7 was significantly higher in the ELT arm (78.6% versus 57.1%; *P* = .035), although salvage rates were similar at days 15, 30 and 60 (Table 4). The median catheter survival duration was longer in the ethanol group (15 versus 8 days; *P* = .118). Recurrence rates by day 60 were significantly lower in the ELT arm (20.5% versus 53.7%; *P* = .002). Mortality was low and comparable between arms (one death in each group by day 30).

**Table 4:** Outcomes of catheter salvaged, mortality and recurrence between the ELT and control arms.

	Catheter salvaged				Mortality				Recurrence			
Follow-up	Total	ELT arm (*n* = 42)	Control arm (*n* = 42)	*P*-value	Total	ELT arm (*n* = 42)	Control arm (*n* = 42)	*P*-value	Total	ELT arm (*n* = 42)	Control arm (*n* = 42)	*P*-value
Day 7	57 (67.86)	33 (78.57)	24 (57.14)	.035^a^	1 (1.19)	0 (0)	1 (2.38)	1^c^	n/a	n/a	n/a	n/a
Day 14	39 (46.99)	20 (48.78)	19 (45.24)	.746^a^	0 (0)	0 (0)	1 (2.38)	1*	7 (2.41)	5 (12.8)	2 (4.76)	.494^c^
Day 30	36 (43.37)	19 (46.34)	17 (40.48)	.59^a^	(2.4)	1 (2.38)	1 (2.38)	1^c^	8 (9.64)	3 (7.32)	5 (11.90)	.713^c^
Day 60	32 (40)	17 (43.59)	15 (36.59)	.523^a^	3 (3.57)	1 (2.38)	2(4.76)	.6^a^	30 (37.50)	8 (20.51)	22 (53.66)	.002^a^

Values presented as n (%).

a Chi-squared test.

b Mann–Whitney test.

c Fisher’s exact test.

Catheter salvage was significantly higher in the ELT arm at day 7 (*P* = .035), but the difference between groups decreased over time. The recurrence rate at day 60 was significantly lower in the ELT group (20.51%) compared with the control group (53.66%; *P* = .002), indicating a protective effect against reinfection. Mortality rates remained low and comparable between groups throughout the follow-up period.

Catheter salvage rates (see Supplementary Table 2) varied depending on the infecting organism, with the highest salvage observed in sterile cases (71.43% in the ELT group versus 66.67% in the control group; OR 1.25) and lowest in *Pseudomonas* infections (0% in the ELT group versus 20% in the control group; OR 0.25). Salvage rates were comparable for CONS (46.67% versus 42.86%; *P* = .837) and *Klebsiella* infections (28.57% versus 16.67%; *P* = 1.0), while *S. aureus* infections had a numerically higher salvage rate in the ELT group (66.67% versus 28.57%) but did not reach statistical significance (*P* = .5, OR 5.0).

The resistance profile of key organisms isolated in culture highlights major concerns regarding antibiotic resistance among CONS, *S. aureus, Klebsiella* and *Pseudomonas. S. aureus* exhibited particularly high resistance to beta-lactams (63.64%), glycopeptides (45.45%) and clindamycin (45.45%). *Pseudomonas* displayed the highest overall resistance rates, particularly to cephalosporins (70%), aminoglycosides (70%) and fluoroquinolones (70%), indicating severe limitations in treatment options. *Klebsiella* also showed high resistance to aminoglycosides (56.25%) and beta-lactams (37.5%). In contrast, CONS infections demonstrated lower resistance rates across most antibiotic classes, making them more susceptible to treatment (Supplementary Table 3).

**Figure 3:**
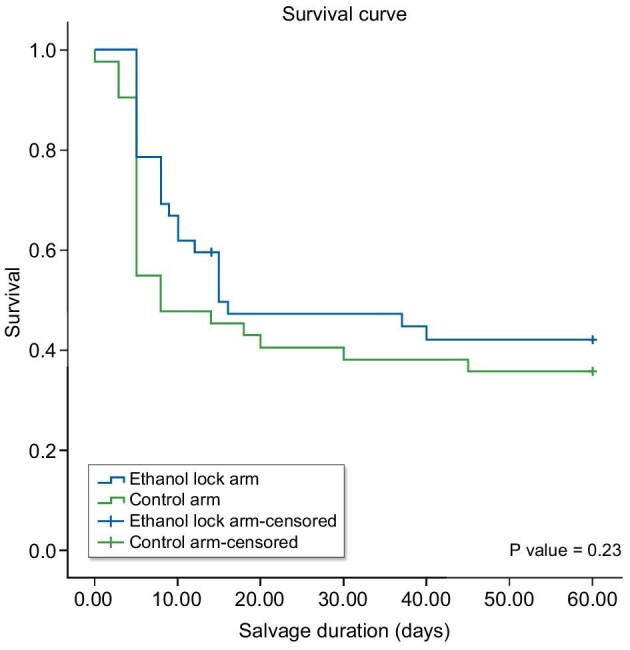
Kaplan–Meier survival analysis curve comparing salvage rate between the ELT arm and control arm.

Kaplan–Meier analysis showed numerically higher catheter survival in the ethanol lock arm at all time points, although differences did not reach statistical significance (logrank *P* = .283). The median survival was 15 days in the ELT group versus 8 days in the control group (Table 5, Fig. 3).

**Table 5:** Kaplan–Meier survival analysis curve comparing the salvage rate between the ELT arm and control arm.

	ELT arm			Control arm		
Time (days)	At risk	Events	Survival probability	At risk	Events	Survival Probability
0	42	0	1	42	0	1
7	42	9	0.786	42	18	0.548
15	33	13	0.496	24	5	0.452
30	20	1	0.471	19	2	0.381
60	19	2	0.422	17	2	0.357
Total (*N*)	42	34		42	27	
Median survival (days)	15			8		
95% CI (days)	0–40.58			3.24–12.76		
Standard error	13.05			2.43		
Logrank *P*-value	0.283					

Multivariate logistic regression showed that higher serum albumin (OR 2.24, *P* = .026) was associated with better salvage, whereas longer dialysis vintage (OR 0.80, *P* = .023) and *Pseudomonas* infection (OR 0.16, *P* = .035) predicted failure (Table 6). Factors like the catheter site were not significant.

**Table 6:** Univariate and multivariate logistic regression to assess significant risk factors of catheter salvage.

	Univariate analysis				Multivariate analysis			
Variable	Beta coefficient	Standard error	*P*-value	OR (95% CI)	Beta coefficient	Standard error	*P*-value	OR (95% CI)
Age (years)	−0.003	0.015	0.861	0.997 (0.968–1.028)				
Number of catheters	−0.153	0.221	0.49	0.858 (0.556–1.324)				
Leucocyte count (cells/µl)	0	0	0.281	1.000 (1.000–1.00)				
C-reactive protein (mg/l)	−0.049	0.03	0.107	0.953 (0.898–1.010)				
Albumin (g/dl)	0.805	0.362	0.026	2.237 (1.099–4.553)	0.889	0.429	0.038	2.432 (1.050–5.633)
Dialysis vintage (months)	−0.123	0.049	0.023	0.802 (0.619–0.992)	−0.104	0.049	0.035	0.902 (0.819–0.992)
PCT (ng/ml)								
5–10	0.448	1.235	0.717	1.566 (0.139–17.631)				
>10	0.041	0.766	0.957	1.042 (0.232–4.681)				
Male	−0.74	0.486	0.128	0.477 (0.184–1.237)				
Comorbidities								
Diabetes mellitus	1.963	2.799	0.483	7.118 (0.029–17)				
Hypertension	0.987	2.798	0.724	2.682 (0.011–64)				
Other	0.79	2.855	0.782	2.204 (0.008–593)				
Prior CRBSI	0.053	0.481	0.913	1.054 (0.410–2.7)				
CONS	0.405	0.712	0.56	1.5 (0.37–6.05)				
*Staphylococcus aureus*	−0.364	0.944	0.699	0.69 (0.10–4.42)				
*Klebsiella*	−1.102	0.717	0.124	0.33 (0.08–1.35)				
*Pseudomonas*	−1.83	0.873	0.035	0.16 (0.02–0.88)	−3.009	1.22	0.014	0.049 (0.005–0.540)

Adverse events were infrequent. Mild flushing was noted in five cases of ELT patients at baseline, with no events in the control group. One case each of fever and flushing occurred by day 7 in the ethanol arm. No adverse events were reported thereafter.

## Discussion

This is the first randomized controlled trial to evaluate the efficacy of ELT in combination with IV antibiotics versus IV antibiotics alone for the salvage of infected tunnelled HD catheters. Catheter salvageability was significantly higher in the ELT group on day 7, but this benefit diminished after the initial week, with no significant differences observed in long-term catheter survival.

Our cohort showed a female predominance (61.9%), differing from other Indian studies that observed a male predominance [11, 12]. The higher female representation in our study reflects a fistula-first policy, with tunnelled catheters reserved for patients with poor vascular access, as observed in prior data from our centre.

The CRBSI rate in our centre was 6.21 per 1000 catheter days, exceeding the recommended benchmark of 3.5 per 1000 catheter days [13]. While lower CRBSI rates of 1.1–1.5 per 1000 catheter days have been reported in Western literature, Indian rates typically range between 5.37 and 6.5 per 1000 catheter days [12, 14–17]. A previous study from our centre reported an even higher CRBSI rate (15 per 1000 catheter days), possibly due to a smaller sample size or broader CRBSI definition [11]. As a tertiary, government-funded institute, factors like overcrowding, poor hygiene, malnutrition and low health literacy likely contributed to the elevated infection rate in our unit.

The median patient age (50.5 years) was lower than reported in many international CRBSI studies [18–22]. Diabetic kidney disease and chronic glomerulonephritis were the leading causes of end-stage renal disease, consistent with global epidemiology.

Fever resolution by day 7 was significantly faster in the ELT group (72.89% versus 35.71%), suggesting improved early symptom control similar to the antibiotic lock study [23]. The dominant organisms were CONS, *Klebsiella pneumoniae, S. aureus* and *Pseudomonas aeruginosa*, with 15.48% sterile culture as noted in Western cohorts [24]. Our MRSA rate (30%) was also in line with their data. Our cohort showed a greater prevalence of Gram-negative organisms, consistent with the Gram-negative predominance in Indian and Western studies and similarly reported worse outcomes in *Pseudomonas*-related CRBSIs despite catheter removal [5, 12, 15, 25–27]. *Pseudomonas* infection was independently associated with poor catheter salvage, probably due to strong biofilm-forming potential and intrinsic resistance mechanism [28–30]. Our multidrug resistance rate (28%) was higher than that reported, likely reflecting regional resistance patterns and antibiotic exposure [31, 32].

The median catheter dwell time prior to CRBSI was 120 days, longer than those reported in Indian studies, possibly due to improved surveillance and earlier detection [12, 15].

The overall salvage rate in the ethanol arm (43.59%) was lower than those reported by Gang *et al.* [5] and Tayebi *et al.* [22], potentially due to higher resistance rates and greater disease severity. Salvage rates with antibiotics alone were comparable to but lower than various studies, ranging from 60 to 80% [9]. Recurrence at 60 days was higher than in both studies, indicating persistent biofilm-related infection. This suggests that ethanol provides local sterilization but offers no added benefit beyond 7 days.

The absolute risk reduction for catheter salvage at day 7 was 21.5%, yielding a number needed to treat (NNT) of ≈5, meaning one additional catheter was salvaged for every five patients treated with ethanol lock. Similarly, the absolute reduction in recurrence by day 60 was 33.2%, with an NNT of ≈3, reflecting a robust protective effect. Although median catheter survival increased by 7 days in the ethanol arm, this difference was not statistically significant.

The mean catheter survival in our cohort was 31 days, shorter than that reported by Gang *et al.* [5] and Tayebi *et al.* [22], likely due to an earlier and aggressive catheter removal protocol in response to complications and, in our case, infective endocarditis and septic shock occurred only in the control group.

Multivariate analysis identified higher serum albumin as an independent predictor of successful catheter salvage, reflecting albumin’s immunomodulatory role [32–35]. In contrast, *Pseudomonas* infection and longer dialysis vintage decreased salvageability. The negative association between dialysis vintage and salvage was also seen in a Pakistani study [36].

ELT was well tolerated, with only minor, transient side effects like flushing and low-grade fever reported in a few cases. No major adverse events occurred beyond day 7. These safety findings are consistent with those reported in the Healthy Cath Trial and other studies [4, 5].

This study shows that ELT can be a cheaper and effective option compared with antibiotic lock therapy for salvaging infected tunnelled dialysis catheters. However, this protocol is feasible primarily in hospitalized patients, as the ethanol lock requires daily instillation over the first week, which is not practical for outpatients who may not be able or willing to travel daily to the dialysis unit. Unlike previous studies, we also looked at predictors of catheter salvage and found that higher serum albumin was a positive factor, while longer dialysis vintage and *Pseudomonas* infection were associated with poorer outcomes. These findings are important because they can help guide which patients are more likely to benefit from salvage attempts and add to the growing evidence supporting ethanol locks, especially in settings where cost and resistance are major concerns.

### Limitations

This was a single-centre, open-label study with a modest sample size, so it may have been underpowered to detect differences in long-term catheter survival. The study was open-label and thus subject to potential observer bias. However, blinding was not feasible given the nature of the intervention involving ethanol lock administration. Although ELT showed a clear benefit in early salvage and recurrence reduction, the primary outcome over 60 days did not reach statistical significance. However, the lower recurrence suggests ethanol may help with local sterilisation of the catheter, which is clinically meaningful. We also included patients with symptoms suggestive of CRBSI but sterile cultures, which reflects real-world practice but may have diluted the observed effect. We acknowledge retrospective Clinical Trials Registry of India registration as a study limitation. The registration was completed after enrolment began due to administrative and logistical constraints but without any change to the study design, objectives or endpoints.

Despite these limitations, the study provides useful data, especially on predictors of salvage, and adds to the growing body of evidence supporting ethanol lock as a practical and cost-effective salvage option.

## Conclusion

This randomized controlled trial demonstrates that ELT, when combined with IV antibiotics, significantly improves early catheter salvage and reduces recurrence rates in tunnelled catheter–related bloodstream infections, with minimal adverse effects. While the advantage in long-term catheter survival was not statistically significant, the early clinical gains support the integration of ELT into salvage protocols, particularly in high-risk or resource-limited settings. Further multicentre studies are warranted to optimize ethanol lock regimens and establish standardized clinical guidelines.

## Supplementary Material

sfag013_Supplemental_File

## Data Availability

The data underlying this article will be shared upon reasonable request to the corresponding author.
