# Supplementary material for: Ethanol lock therapy for salvage of infected tunnelled haemodialysis catheters: a randomised controlled trial
Source: Clin Kidney J. 2026 Jan 19;19(3):sfag013. doi: 10.1093/ckj/sfag013 (PMC12950961; doi:10.1093/ckj/sfag013)
Supplement: sfag013_Supplemental_File [file sfag013_supplemental_file.docx]

**Supplementary Table 1: -Comparison of baseline biochemical and culture parameters between Ethanol lock and control arm.**

| **Baseline biochemical parameters** | **Total** | **Ethanol lock arm(n=42)** | **Control arm(n=42)** | **P value** |
| --- | --- | --- | --- | --- |
| **Procalcitonin (ng/mL)** | | | | |
| 2-5 | 8 (9.52%) | 1 (2.38%) | 7 (16.67%) | 0.032^*^ |
| 5-10 | 4 (4.76%) | 1 (2.38%) | 3 (7.14%) |  |
| >10 | 72 (85.71%) | 40 (95.24%) | 32 (76.19%) |  |
| **Hemoglobin (g/dL)** | 8.15(7.6-8.8) | 7.8(7.6-8.6) | 8.45(7.5-8.8) | 0.882^‡^ |
| **Leukocyte count (cells/µL)** | 18500(11900-23000) | 20000(12525-23000) | 16850(11500-23100) | 0.764^‡^ |
| **Platelet count (cells/µL)** | 2.45(1.475-3.2) | 2.6(1.6-3.425) | 1.8(1.4-3.2) | 0.145^‡^ |
| **C-reactive protein (mg/L)** | 11(6.675-12) | 8.65(6.425-12.75) | 11(7.65-12) | 0.379^‡^ |
| **Albumin (g/dL)** | 3.3(2.6-3.6) | 3.3(2.6-3.6) | 3.3(2.625-3.6) | 0.832^‡^ |

**^‡^ Mann Whitney test, ^*^ Fisher's exact test. Baseline biochemical and inflammatory markers were comparable between the two groups, with no significant differences in hemoglobin, leukocyte count, platelet count, C-reactive protein, and albumin levels. However, procalcitonin levels were significantly higher in the control arm, with a greater proportion of patients exhibiting moderate elevations (P = 0.032). This finding suggests that the control group had a higher baseline inflammatory burden.**

**II CRBSI Rate Calculation**

**Total number of catheters inserted during the period were 188, out of which 95 presented with CRBSI with median catheter days of 120 days (IQR 60-180). CRBSI rate = Number of CRBSI / Total number of catheter days.The CRBSI rate is 6.21 per 1000 catheter days, with a standard deviation (SD) of 3.12**

**Supplementary Table 2: Complications of Catheter related blood stream infection**

| **Complications** | **Ethanol lock arm(n=42)** | **Control arm(n=42)** | **Total (n=84)** | **P value** |
| --- | --- | --- | --- | --- |
|  |  |  |  | 1^*^ |
| Exit site Discharge | 1(2.38%) | 2 (4.76%) | 3 (3.57%) |  |
| Tunnel site infection | 3 (7.14%) | 3 (7.14%) | 6 (7.14%) | 0.32 |
| Infective endocarditis | 0(0%) | 2 (4.76%) | 2(2.38%) | N/A |
| Septic Shock | 0(0%) | 1(2.38%) | 1(1.19%) | N/A |
| Pneumonia | 3 (7.14%) | 6(14.28%) | 9(10.71%) | 0.4729 |
| Pleural effusion | 3 (7.14%) | 1(2.38%) | 4(4.76%) | 0.6359 |

**Chi square test**

**Supplementary table 2: Comparison of salvage rate in various organisms between Ethanol lock and control arm.**

| **Organisms** | **Total (n=84)** | **Ethanol lock arm(n=42)** | **Control arm(n=42)** | **P value** | **Odds ratio (95% CI)** |
| --- | --- | --- | --- | --- | --- |
| Sterile | 9 /13 (69.23%) | 5 /7 (71.43%) | 4/6 (66.67%) | 1^*^ | 1.250 (0.118 to 13.240) |
| Coagulase negative staphylococcus | 13 /29 (44.83%) | 7/15 (46.67%) | 6/14 (42.86%) | 0.837^†^ | 1.167 (0.269 to 5.054) |
| Staphylococcus aureus | 4 /10 (40%) | 2/3  (66.67%) | 2/7  (28.57%) | 0.5^*^ | 5 (0.273 to 91.518) |
| Klebsiella | 3 /13 (23.08%) | 2/7  (28.57%) | 1/6  (16.67%) | 1^*^ | 2 (0.134 to 29.808) |
| Pseudomonas | 1/10  (10%) | 0/5  (0%) | 1/5  (20%) | 1^*^ | 0.250 (0.006 to 10.536) |
| Enterobacter | 1 /1 (100%) | 1/1  (100%) | 0 (0%) | NA | - |
| E coli | 0/1 (0%) | 0 (0%) | 0 (0%) | NA | - |
| Citrobacter | 1 /1 (100%) | 0 (0%) | 1/1  (100%) | NA | - |
| Acinetobacter | 0/2 (0%) | 0 (0%) | 0 (0%) | NA | - |

**^*^ Fisher's exact test, ^†^ Chi square test**

The resistance profile of key organisms isolated in culture highlights major concerns regarding antibiotic resistance among Coagulase-negative Staphylococcus (CONS), Staphylococcus aureus, Klebsiella, and Pseudomonas. MRSA rates were 30%, Carbapenem Resistant organism were around 20-30%.

**Supplementary table 3: Resistance profile of Key organisms isolated in culture**

| **Antibiotics** | **Cons Resistance (%)** | **Staph aureus Resistance (%)** | **Klebsiella Resistance (%)** | **Pseudomonas Resistance (%)** |
| --- | --- | --- | --- | --- |
| Beta-Lactam | 4 (13.79%) | 7 (63.64%) | 3 (37.5%) | 5 (50%) |
| Glycopeptide | 2 (6.9%) | 5 (45.45%) | n/a | n/a |
| Cephalosporin | 4 (13.79%) | 4 (36.36%) | 3 (37.5%) | 7 (70%) |
| Linezolid | 2 (6.9%) | 2 (18.18%) | n/a | n/a |
| Carbapenem | n/a | n/a | 2 (25%) | 2 (20%) |
| Colistin | n/a | n/a | 2 (25%) | 1 (10%) |
| Aminoglycoside | 6 (20.69%) | 1 (9.09%) | 4 (56.25%) | 7 (70%) |
| Clindamycin | 2 (6.9%) | 5 (45.45%) | n/a | n/a |
| Fluoroquinolone | 4 (13.79%) | 1 (9.09%) | 3 (37.5%) | 7 (70%) |
|  |  |  |  |  |

**Resistance patterns varied by pathogen, with Staphylococcus aureus showing the highest resistance rates to beta-lactams (63.64%) and glycopeptides (45.45%), while Pseudomonas exhibited extensive resistance to cephalosporins (70%) and aminoglycosides (70%). The higher resistance rates in the control arm suggest that ethanol lock therapy may contribute to better infection control.**
